# Supplementary material for: Prevalence and associated risk factors of burnout amongst veterinary students in Ghana
Source: PLoS One. 2022 Jul 14;17(7):e0271434. doi: 10.1371/journal.pone.0271434 (PMC9282513; doi:10.1371/journal.pone.0271434)
Supplement: Attachment — Submitted filename: Burnout Mansucript with Edits.pdf [file pone.0271434.s002.pdf]

PREVALENCE AND ASSOCIATED RISK FACTORS OF BURNOUT AMONGST VETERINARY STUDENTS IN GHANA

[1]Benjamin Obukowho Emikpe
[2]Derrick Adu Asare, [1]Ludwig Albert Nortey Botchway, [3]Abigael Omowumi Emikpe and [1]Richard Abeiku Bonney.

[1] *School of Veterinary Medicine, Kwame Nkrumah University of Science and Technology, Kumasi, Ghana*
[2] *University of Ghana, Legon, Accra, Ghana*
[3] *Department of Nursing, Faculty of Allied Health Sciences, Kwame Nkrumah University of Science and Technology, Kumasi, Ghana.* Email: benabis2001@gmail.com

*Corresponding author: Professor Benjamin Emikpe, *School of Veterinary Medicine, Kwame Nkrumah University of Science and Technology, Kumasi, Ghana*
Telephone: +233549410841 Email: banabis2001@yahoo.com,

ABSTRACT

This study was designed as a cross-sectional study at Kwame Nkrumah University of Science and Technology (KNUST) in Kumasi – Ghana to find out the prevalence and associated risk factors of burnout among veterinary students. A total of 74 veterinary students served as the respondents and were ~~fed with~~ given online questionnaires which comprised questions on emotional exhaustion (EE), depersonalisation (DP) and reduced personal accomplishment (RPA). Data obtained were analysed using descriptive statistics, Chi-square test and regression analysis. Results indicated that ~~averagely~~ on average, the EE and RPA were low with mean score of 12.72 ± 4.46 and 27.96 ± 7.94 whilst DP were high with a mean score of 20.72 ± 6.5 among veterinary students. The year of study had a significant effect (p=0.000) on burnout with levels of high RPA (80%) and DP (70%) being more in the preclinical students as compared to the clinical students (RPA= 20%, DP= 30%). There was also a significant effect (p=0.028) of the year of type of residence of the students on the high level of DP and RPA. Daily sleep hours of the veterinary students were also indicated as a significant associated risk factor of burnout among veterinary students in this study as more less sleep hours

(<6hours) causes high levels of DP and RPA. Age, gender and marital status, working status, exercise hours and weekly study hours of students also did not count as significant predictive factors ($p>0.05$) of burnout.

Keywords: Burnout, Veterinary students, Depersonalization, Emotional Exhaustion

INTRODUCTION

Background

Medicine related programmes worldwide such as human medicine is aimed at training and developing world-class students to be well-equipped with the required skills and attitudes necessary to ensure the health needs of immediate societies and the world at large are not left in jeopardy [1]. In view of this focus, medical schools across the world have focused on developing a more robust curriculum and nature of studies for their students so that no stone will be left unturned in the bid to delivering good quality medical school education [2] of which veterinary medical schools are no exceptions. The robustness of such medical schools can be very stressful for students and can cause burnout syndrome.

Burnout syndrome is characteristically a combination of negative energies that are manifested in emotional and physical exhaustion, depersonalisation, and reduced personal achievements [3]. The burnout syndrome has been reported the more in professionals and workers as well as students in the human medical schools [4,5] with less report on veterinary students. In the academic setting, burnout has been reported by several researchers to be occurring in students of various universities in different countries [6,7,8] of which it has been found that veterinary students are no exception to being at risk of it [9,10,11]. Research has indicated strongly that enrolling in a veterinary school

comes with a certain level of exposure to stress. This kind of stress exposure is mostly prolonged due to the lengthy years spent in training as a veterinary student [9]. These stresses could arise from many sources which could be either academic related or non-academic related. Academically, the requirement of universities and the demand on students to maintain very good academic standings to keep them in the school, coupled with the heavy workload of the veterinary medicine curriculum causes a relatively high level of stress amongst students [12,13]. In addition to the demanding curriculum, the amount of time dedicated to learning the vast information on the numerous animal species is another source of academic stress [14]. The non- academic sources of stress include the perceived difficulty in fitting in as well as unclear expectations on the part of students, poorer perceived physical health, financial stress and certain stressful activities that students may be involved in during their course of study among others [15,16,17]. These stresses on students when in excess leads to the burnout syndrome and can definitely affect the academic performance of students.

Though several research works have been reporting burnout among students in the medical and veterinary medical schools, the review of literature indicated a lack of studies in the burnout amongst veterinary students in West Africa. There is therefore the need to fill this gap in literature concerning burnout amongst veterinary students in Ghana. This study therefore is focused on examining the level of burnout amongst veterinary students in Ghana as well as the risk factors that exposes veterinary students in Ghana to the burnout syndrome. The findings of this study will be very key in informing the stakeholders in veterinary education to take pragmatic and proactive measures to deal with burnout syndrome amongst veterinary students to enhance their academic well-being.

METHODS

Research Design and Study Area

This study was designed as a cross-sectional study at the School of Veterinary Medicine (SVM) in Kwame Nkrumah University of Science and Technology (KNUST) in Kumasi - Ghana. This research was conducted within a month between June and July 2020.

The School of Veterinary Medicine is a faculty under the College of Health Sciences in Kwame Nkrumah University of Science and Technology in Kumasi; the capital city of Ashanti Region of Ghana. The school has its campus at Boadi, a suburb of Kumasi. The veterinary programme run by the School of Veterinary Medicine is a 6-years programme with students in first, second and third years of study classified as pre-clinical students whilst students in clinical years are those in fourth, fifth and sixth years of study. The School does not have a residence area on campus for students and in view of this, students bear the responsibility to search for accommodation which could be far or near.

Study Population

The study population in this current research comprised all the students of the School of Veterinary Medicine in Kwame Nkrumah University of Science and Technology. The overall population of the students in the School of Veterinary Medicine as at the time of data collection for this study stood at a total of one hundred and twenty-five (125), in different years of study. Characteristically, the student population comprises of different individuals with different socio-economic backgrounds and from different ethnicities including some international students from some African countries including Sierra Leone, Burkina Faso, Nigeria and Uganda.

Sampling technique and sample size

The sampling technique employed for this study was the simple random sampling technique. This method of sampling technique was used so as to avoid biases on the part of the researcher as well as to ensure that each student was provided with the chance of being selected to be a part of the respondents for this study.

In determining the sample size, the Yamane formula [18] was used. The Yamane formula is stated as $n = N/1+N\,(e)^2$; where n is sample size. N is population size, e is sampling error constant, power of constant which is 0.05. For sample size calculation;

$n = ?,\quad N = 125,\ e = 0.05$

Therefore, $n = 125\ /\ [1+125\ (0.05)^2]$

$n = 125\ /\ [1+ 125(0.0025)]$

$n = 125\ /\ [1+ 0.3125]$

$n = 125\ /\ 1.3125$

$n = 95$

A total of 95 students were needed for the study based on the above calculation. Nevertheless, due to COVID and technological constraints because of the lockdown and some of the students had limited access to internet connection, a total of 74 students were able to access and fill the online questionnaires administered. These student respondents were used as the sample size for this study. How do the researchers rectify the difference btw the calculation from the Yamane formula and the actual sample?

Data collection instrument and Procedure

In this study, a well-structured questionnaire was designed with Google Forms which served as the data collection instrument. The questionnaire comprised questions on the demographic characteristics of the respondents as well as certain practices such as their sleeping hours, exercise hours and study hours. The other section of the questionnaire comprised of questions on emotional exhaustion (EE), Depersonalisation (DP) and Reduced Personal Accomplishment (RPA). Question on EE ~~bothered~~ focused on the feeling of being emotionally and physically drained as a result of the workload of a veterinary student. Additionally, questions on DP ~~bothered~~ focused on ~~negative and feeling of indifference to~~ feelings of negativity and indifference towards others whilst questions on RPA ~~bothered~~ focused on the students' personal unfavourable judgement of themselves in terms of their academic performance.

The questionnaires were successfully administered to the respondents after obtaining their consent. The link to the questionnaires to be completed were delivered to the respondents through electronic platforms (specifically WhatsApp). The responses to the questionnaires were retrieved from Google Forms in a Microsoft Excel format by the researcher after the one-month (from June to July, 2020) time frame had elapsed.

Data Analysis

In this study, data analysis performed on all the data collected were performed using Statistical Package for Social Sciences (SPSS Version 20). Descriptive statistics were used to analyse the demographic characteristics (age, gender, year of study, marital status and residence) and practices of students as well as the responses of the students on emotional exhaustion, depersonalisation and

reduced personal accomplishment in the form of frequencies, percentages, mean and standard deviations. Pearson Chi-square test was used to determine the significant association between categorical variables which were grouped.

With respect to the effect of demographic characteristics and practices of students on the burnout of students, the multiple logistic regression analysis was used. The dependent variable was a high combined score on subscales of DP and RPA. Independent variables included all the demographic characteristics and practices of the students. The statistical significance was tested at 5% significance level (p value = 0.05).

RESULTS

Demographic Characteristics of Students

Results obtained for the demographic characteristics of the respondents from the data analysis are displayed in Table 1 below.

The findings indicated that out of the 74 respondents, 41 (55.4%) respondents were less than 22 years of age whilst the remaining 33 (44.6%) of the respondents were above 22 years of age. With respect to the gender distribution of the respondents, 54 (73%) of the respondents were males whilst the remaining 20 (27%) of the respondents were females. In terms of marital status of the respondents, 69 (94.6%) of the respondents were unmarried whilst the remaining 5 (5.4%) of the respondents were married. The level of study of the respondents was also considered in this study as shown in Table 1 below. The findings showed that 31 (41.9%) of the respondents were first year students, 7 (9.5%) of the respondents were second year students, 14 (18.9%) of the respondents were third year students, 10 (13.5%) were fourth year students, 9 (12.2%) of the

respondents were fifth year students whilst the remaining 3 (4.1%) of the respondents were sixth year students. Results on the place of residence of veterinary students showed that 41.9% were living in rented apartments, 35.1% were living in dormitories, 12.2% were living with their parents / families, 8.1% were living in hostels whilst 2.7% were living in traditional halls.

Table 1. Results of demographic characteristics of respondents

Variable	Category	Frequency (n)	Percentage (%)
Age of Students	<22	41	55.4
	>22	33	44.6
Gender of Students	Female	20	27.0
	Male	54	73.0
Marital Status of Students	Married	5	5.4
	Not Married	69	94.6
Year of Study	Preclinical (Year 1- Year 3)	52	70.3
	Clinical (Year 4-Year 6)	22	29.7
Residence of Students	Dormitory	26	35.1
	Hostel	6	8.1
	Rented apartment	31	41.9
	Traditional Hall	2	2.7

	With parent /family	9	12.2

Practices of Students

Findings on the practices of veterinary students were considered in this study as shown in Table 2 below. Results on the weekly study hours showed that 50% studied between 25-40 hours a week, 31.1% studied less than 25 hours a week whilst 18.9% studied more than 40 hours in a week. It was observed that with respect to exercise hours amongst veterinary students, 64.9% spent less than 2 hours exercising, 28.4% spent between 2-5 hours exercising whilst 6.8% exercised above 5 hours. With sleep hours of students, majority of the students (62.2%) slept less than 6 hours whilst 37.8% slept between 6-10 hours. It was also indicated that 87.8% of the students were not working as they study whilst the remaining 12.2% were engaged in diverse forms of work alongside studying.

Table 2. Results of practices of respondents

Variable	Category	Frequency (n)	Percentage (%)
	Less than 25 hours	23	31.1
Weekly Study Hours	Between 25-40 hours	37	50.0
	Above 40 hours	14	18.9
	Less than 2 hours	48	64.9
Exercise Hours	Between 2-5 hours	21	28.4

	Above 5 hours	5	6.7
Sleep Hours	Less than 6 hours	46	62.2
	Between 6-10 hours	28	37.8
Works whiles studying	Not Working	65	87.8
	Working	9	12.2

Prevalence of Burnout Amongst Students

Table 3 below summarizes the prevalence of burnout amongst veterinary students. It was observed that majority of the students (86.5%) students reported low levels of emotional exhaustion whilst 13.5% reported moderate levels of emotional exhaustion. Majority of the students (94.6%) reported a high level of depersonalization whilst 5.4% of the students reported moderate level of depersonalization ~~respectively~~. Moreover, majority of the students ~~represented by 77.0% of the students~~ showed low levels of reduced personal accomplishment with 16.2% and 6.8% of the students showed moderate to high levels of reduced personal accomplishment as shown in Table 3 below.

The mean scores for the various components of burnout indicated that on ~~the~~ average, the emotional exhaustion of the students were low with mean score of 12.72 ± 4.46. Depersonalization levels of the students in this study were high with a mean score of 20.72 ± 6.51. With reduced personal accomplishment, the mean score of 27.96 ± 7.94 indicated that the students were satisfied with their personal accomplishments as shown in Table 3 below. It can be inferred from the results

that depersonalization is the major contributing component to burnout in the veterinary students considered in this study.

Table 3. Results on the prevalence of burnout amongst respondents

Variable	Categories / Levels	Distribution (n (%))	Mean subscales score
	Low (≤17)	64 (86.5%)	12.72 ± 4.46
Emotional Exhaustion (EE)			
	Moderate (18-29)	10 (13.5%)	
Depersonalization (DP)	Moderate (6-11)	4 (5.4%)	
			20.72 ± 6.51
	High (≥12)	70 (94.6)	
	Low (≤33)	57 (77.0%)	
Reduced Personal Accomplishment (PA)	Moderate (34-39)	12 (16.2%)	27.96 ± 7.94
	High (≥40)	5 (6.8%)	

RISK FACTORS OF BURNOUT

Demographic Characteristics and Burnout

The prevalence of burnout amongst the veterinary students in this study with respect to the demographic characteristics of the respondents was compared as summarized in Table 4 below. With respect to emotional exhaustion (EE), no student indicated high EE. For age, majority in both age groups of students indicated low EE whilst 7 (70%) of the students who indicated moderate EE were above 22 years of age. More females 6 (60%) indicated moderate levels of EE than males 14 (40%). More students (7, 70%) in the preclinical year reported moderate levels of EE as

compared to the clinical year students (3, 30%). More students in rented apartments (50%) reported moderate levels of EE as compared those living in dormitories (10%), traditional halls (0%), hostels (10%) and staying with their parents/ family (20%) Table 4.

Table 4: Results on demographic characteristics and burnout levels

		**Emotional Exhaustion (EE)**		**Depersonalization (DP)**		**Reduced Personal Accomplishment (RPA)**		
**Characteristic**	**Category**	**Low**	**Moderate**	**Moderate**	**High**	**Low**	**Moderate**	**High**
Age (years)	<22	38 (59.4%)	3 (30%)	1 (25%)	40 (57.1%)	33(54.4%)	4 (33.3%)	4 (80%
	>22	26 (40.6%)	7 (70%)	3 (75%)	30 (42.9%)	24(45.6%)	8 (66.7%)	1 (20%
Gender	Male	50 (78.1%)	4 (40%)	2 (50%)	52 (74.3%)	50(87.7%)	3 (25%)	2(41.6%
	Female	14 (21.9%)	6 (60%)	2 (50%)	18 (25.7%)	8(59.4%)	9 (75%)	3(59.4%
Marital Status	Married	4 (6.3%)	1 (10%)	3 (75%)	2 (2.9%)	4 (7%)	0 (0%)	0 (0%)
	Single	60 (93.7%)	9 (90%)	1 (25%)	68 (97.1%)	53 (93%)	12 (100%)	5(100%
Year of study	Preclinical	45 (70.3%)	7 (70%)	3 (75%)	49 (70%)	38(66.7%)	10 (83.3%)	4 (80%
	Clinical	19 (29.7%)	3 (30%)	1 (25%)	21 (30%)	19(33.3%)	2 (16.7%)	1 (20%
Residence	Dormitory	25 (39.1%)	1 (10%)	2 (50%)	24 (34.3%)	23(40.3%)	3 (25%)	0 (0%)
	Parents /family	7 (10.9%)	2 (20%)	0 (0%)	9 (12.9%)	3 (5.3%)	2 (16.7%)	4 (80%

Rented apartment	26 (40.6%)	5 (50%)	0 (0%)	31 (44.3%)	25 (43.8%)	5 (41.7%)	1 (20%
Traditional Hall	2 (3.1%)	0 (0%)	1 (25%)	1 (1.4%)	1 (1.8%)	1 (8.3%)	0 (0%)
Hostel	4 (6.3%)	2 (20%)	1 (25%)	5 (7.1%)	5 (8.8%)	1 (8.3%)	0 (0%)

Considering depersonalization, more students who were less than 22 years (40, 57.1%) reported high levels of DP. It was observed that more male students (52, 74.3%) reported high DP as compared to 18 (25.7%) of the females. More students (49, 70%) in the preclinical year reported high levels of DP as compared to the clinical year students (21, 30%). It was further observed that more students in rented apartments (44.3%) reported high levels of EE as compared those living in dormitories (34.3%), traditional halls (1.4%), hostels (7.1%) and staying with their parents/ family (12.9%) (Table 4 above). – Were all of these differences statistically significant? More details, including chi-square ~~and~~ stats and p-value, are needed throughout this section

Results on reduced personal accomplishment showed that with respect to high RPA, more students [as compared to?] who were less than 22 years (80%) reported high levels of RPA. It was observed that female students (59.4%) reported high RPA as compared to the males (41.6%). More students (80%) in the preclinical year reported high levels of RPA as compared to the clinical year students (20%). It was also observed that more students living with their families or parents (80%) reported high levels of RPA as compared those living in dormitories (20%). However, with respect to moderate levels of RPA, more students living in rented apartments (41.7%) indicated moderate levels of RPA as compared to students in other types of residence (Table 4 above).

Prevalence of Burnout with respect to practices of students

Results on prevalence of burnout amongst the veterinary students with respect to their practices have been presented in Table 5 below. No student indicated high level of emotional exhaustion (EE).

Table 5: Results on student practices and burnout levels

		**Emotional Exhaustion**		**Depersonalization**		**Reduced Personal Accomplishment**		
**haracteristic**	**Category**	**Low**	**Moderate**	**Moderate**	**High**	**Low**	**Moderate**	**High**
'ork status	Working	7 (10.9%)	2 (20%)	1 (25%)	8 (11.4%)	7(9.7%)	1(8.3%)	1 (20%
	Not working	57 (89.1%)	8 (80%)	3 (75%)	62 (88.6%)	50 (69.4%)	11(91.7%)	4 (80%
Weekly Study hours	< 25 hrs	18 (28.1%)	5 (50%)	0 (0%)	23(32.9%)	16 (28.1%)	4 (33.3%)	3 (60%
	25- 40 hrs	33 (51.6%)	4 (40%)	2 (50%)	32(45.7%)	33 (57.9%)	2 (16.7%)	2 (40%
	> 40 hrs	13 (20.3%)	1 (10%)	2 (50%)	12 (17.1%)	8 (14%)	6 (50%)	0 (0%
ercise Hours	< 2 hrs	42 (65.6%)	6 (60%)	1 (25%)	47(67.2%)	37 (64.9%)	9 (75%)	2 (40%
	2 – 5 hrs	20 (31.3%)	1 (10%)	2 (50%)	19 (27.1%)	16 (28.1%)	3 (25%)	2 (40%
	> 5 hrs	2 (3.1%)	3 (30%)	1 (25%)	4 (5.7%)	4 (7%)	0 (0%)	1 (20%
eep Hours	< 6 hrs	39 (60.9%)	7 (70%)	3 (75%)	43 (61.4%)	36 (63.2%)	7 (58.3%)	3 (60%
	6-10 hrs	25 (39.1%)	3 (30%)	1 (25%)	27(38.6%)	21 (36.8%)	5 (41.7%)	2 (40%

($p>0.05$) of age, gender, marital status on the high levels of depersonalization and reduced personal accomplishments of the veterinary students in this study.

The results also showed that there was found a significant effect ($p<0.05$) of the year of study of the veterinary students as well as the residence of the students on the high level of DP and RPA represented by significance values of $p=0.000$ and $p=0.028$ respectively. The findings show that the type of residence and year of study are significant risk factors of high levels of depersonalization and reduced personal accomplishment among veterinary students.

Table 6: Tabulation of students' demographic characteristics and high levels of DP and RPA

Characteristic	Category	DP	RPA	P-value
Age (years)	<22	40 (57.1%)	4 (80%)	0.204
	>22	30 (42.9%)	1 (20%)	
Gender	Male	52 (74.3%)	2 (41.6%)	0.240
	Female	18 (25.7%)	3 (59.4%)	
Marital Status	Married	2 (2.9%)	0 (0%)	0.860
	Single	68 (97.1%)	5(100%)	
Year of study	Preclinical	49 (70%)	4 (80%)	0.000*
	Clinical	21 (30%)	1 (20%)	
Residence	Dormitory	24 (34.3%)	0 (0%)	0.028*
	Parents /family	9 (12.9%)	4 (80%)	

For depersonalization, the majority of the students (88.6%) who indicated high levels of DP were students who were not working whiles studying. More students (45.7%) who indicated high levels of DP studied between 25-40 hrs a week as compared to those who studied more than 40 hours a week (17.1%) and less than 25 hours a week (32.9%). Furthermore, more students who exercised less than 2 hours weekly (67.2%) indicated high levels of DP as compared to the other students who exercised more than 2 hours weekly (include % here). In addition, more students who indicated high levels of DP slept less than 6 hours in a day (63.2%) as compared to ~~32.8%~~ those who slept between 6-10 hours in a day (32.8%) as shown in Table 5 above.

With reduced personal accomplishment (RPA), the majority of the students (88.6%) who indicated high levels of RPA were students who were not working whiles studying. More students (45.7%) who indicated high levels of RPA studied less than 25 hours a week as compared to those who studied more than 40 hours a week (0%) and between 25 – 40 hours a week (40%). Furthermore, more students who exercised less than 2 hours weekly (40%) and between 2-5 hours (40%) indicated high levels of RPA as compared to the other students who exercised more than 5 hours weekly (%). In addition, more students who indicated high levels of RPA slept less than 6 hours in a day (60%) as compared to ~~40%~~ those who slept between 6-10 hours in a day (40%) as shown in Table 5 above.

Effects of Demographic Characteristics on Burnout – More statistical details are needed here and throughout the results section, including the type of analysis conducted and the specific statistics (e.g., chi-square, correlation coefficient, etc).

Findings on the effects of students' demographic characteristics on high levels of DP, and RPA were displayed in Table 6 below. The findings revealed that there was no significant effect

	Rented apartment	31 (44.3%)	1 (20%)
	Traditional Hall	1 (1.4%)	0 (0%)
	Hostel	5 (7.1%)	0 (0%)

** = p-value is significant at 5% significance level (95% Confidence interval).*

Effects of students practices on burnout

The result on the effect of practices of students on high levels of DP and RPA is displayed in Table 7 below. The results revealed that there were no significant effects ($p>0.05$) of ~~the~~ working status, weekly study hours and exercise hours of the veterinary students on the high levels of depersonalization and reduced personal accomplishments of the veterinary students in this study. Though there were noticeable differences between the categories under each student practice risk factors for high DP and PA, these differences were not significant.

However, there was a statistically significant effect ($p<0.05$) of the sleep hours of students on the high levels of DP and RPA. This finding shows that the sleeping hours of students are significant risk factors of high levels of depersonalization and reduced personal accomplishment among veterinary students.

Table 7: Results on practices and burnout levels

Characteristic	Category	DP	RPA	P-value
Work status	Working	8 (11.4%)	1 (20%)	0.086

	Not working	62 (88.6%)	4 (80%)	
Weekly Study hours	< 25 hrs	23(32.9%)	3 (60%)	0.234
	25- 40 hrs	32(45.7%)	2 (40%)	
	> 40 hrs	12 (17.1%)	0 (0%)	
Exercise Hours	< 2 hrs	47(67.2%)	2 (40%)	0.098
	2 – 5 hrs	19 (27.1%)	2 (40%)	
	> 5 hrs	4 (5.7%)	1 (20%)	
Sleep Hours	< 6 hrs	43 (61.4%)	3 (60%)	0.049*
	6-10 hrs	27(38.6%)	2 (40%)	

** = p-value is significant at 5% significance level (95% Confidence interval).*

DISCUSSION

This study investigated the prevalence and associated risk factors of burnout among veterinary students in Ghana. The outcome of the findings of this study indicated that on the average, the Emotional Exhaustion (EE) of veterinary students was low with mean score of 12.72 ± 4.46. Reduced Personal Accomplishment (RPA) was also low on the average with a mean score of 27.96 ± 7.94 whilst Depersonalisation (DP) levels of the veterinary students in this study were high with a mean score of 20.72 ± 6.5. This finding shows that the level of burnout among veterinary students is not relatively high; however, burnout is expressed the most due to depersonalisation. This finding is in sharp contrast to the findings of a study conducted by McArthur [10] who in his

findings showed that there exists a high level of burnout among veterinary students in Australia. The differences in the prevalence of burnout could be as a result of differences in geographical location as well as the differences in the nature of the veterinary medicine curriculum in these two different countries. This study also revealed that levels of emotional exhaustion experienced by the veterinary students were low to moderate with majority of the students experiencing low levels of emotional exhaustion.

Findings on the effects of demographic characteristics of veterinary students on the high levels of DP and RPA showed that age did not have any significant effect on the high levels of DP and RPA; though the students who were less than 22 years of age recorded high levels of DP (57.1%) and RPA (80%) as compared to the students who were older than 22 years of age (high DP=429%; high RPA = 20%). This shows that burnout amongst veterinary students is not significantly affected by the age of the student though those ~~within the lower~~ age ~~limits~~ suffer the stress of depersonalization and reduced accomplishments ~~the~~ more.

The findings of this current study furthermore showed that there is no significant ~~(p>0.05)~~ impact of gender on the prevalence of burnout among veterinary students although females ~~more frequently~~ reported ~~higher levels of~~ RPA (59.4%) as compared to male students (41.6%) whilst the male students (74.3%) reported ~~higher levels~~ of DP as compared to the females (25.7). This finding corroborates the findings of IlićZˇ ivojinović *et al.*, [9] who reported that though female students reported higher levels of burnout especially with emotional exhaustion, the effect of gender on burnout levels were not statistically significant. Notwithstanding, the findings of this study is in sharp contrast with the findings of Gelberg and Gelberg [19] who in their study on stress in veterinary students established that gender has a significant effect on stress and burnout levels with females being the most susceptible as compared to their male counterparts.

This study revealed that the levels of high RPA (80%) and DP (70%) was more in the preclinical students as compared to the clinical students (RPA= 20%, DP= 30%). This effect of the year of study on the level of burnout experienced by veterinary students in this study was statistically significant. This could be attributed to the fact that students in preclinical years are new to the stressors involved in being a student in veterinary school and hence are now adjusting to the pressures of balancing the rigorous academic course with extracurricular activities. Whereas students in the clinical years have been preconditioned and have adjusted to the academic stress involved in being a veterinary medical student. This finding is in agreement with the findings of Feras *et al.* [20] who in their report concerning burnout in medical students in Lebanon indicated that there is a high level of 75% burnout among preclinical medical students.

The residence of students was found to have significant effect on the prevalence of burnout in this study. Students who were residing in rented apartments (80%) as well as those living with their families (44.3%) recorded higher levels of DPA and RPA respectively as compared to those who lived in halls (1.4%) on the main KNUST campus. This finding could be explained by the mode of transportation of students from their places of residence to the Veterinary school campus which is located quite a distance away from the main KNUST campus. Also, the financial constraint posed to most students in renting apartments, and hostels could explain this effect of place of residence on burnout levels. The findings of this study agrees with the findings of Chigwere *et al* [11] who indicated the type of living arrangements of students in California was associated with burnout.

In this study, the marital status of the veterinary students did not have any significant effect (p>0.05) on the high levels of depersonalization and reduced personal accomplishments of the veterinary students. However, there were noticeable differences with high levels of DP and RPA

recorded more in the unmarried students of 97.1% and 100% respectively. This difference could be as a result of the lack of emotional partners who can support them to cope in times where they feel unhappy about their academic performances as well as have negative thoughts about their programme of study.

Furthermore, this study revealed that some practices of students could serve as risk factors of burnout in their academic pursuit. The findings of the study revealed sleep hours had significant effect on the prevalence of burnout. Veterinary students who slept less than 6 hours in a day in this study indicated high levels of DP (61.4%) and RPA (60%) as compared to those who slept between 6-10 hours in a day (high RPA= 40%; high DP= 38.6%). This phenomenon could be attributed to the fact that sleep is significant in reenergising the body and the mind to deal with academic activities. Therefore, less amount of sleep will result in excessive tiredness of both the body and the mind which will affect academic performance as well as lead to low physical energy to carry-out daily activities effectively and efficiently. I'm not sure that this finding is particularly groundbreaking.

There was also found no significant effects (p>0.05) of the working status, weekly study hours and exercise hours of the veterinary students on ~~the high levels of~~ depersonalisation and reduced personal accomplishments.

CONCLUSION

But you found that Burnout was below average in areas of EE & RPA. Can you provide logic and/or analysis that and support the idea that burnout is expressed through depersonalization?

Burnout was observed among veterinary students in Ghana. This was expressed in high depersonalisation with low levels of emotional exhaustion and low levels of reduced personal achievement. The year of study and the place of residence of veterinary students were found to be the associated demographic risk factors for burnout. Daily sleep hours of the veterinary students

were also indicated as an associated risk factor of burnout among veterinary students in this study. These findings should inform measures that need to be put in place by the university authority to reduce this stressful syndrome.

REFERENCES

1. Altannir Y, Alnajjar W, Ahmad SO, Altannir M, Yousuf F, Obeidat A, Al-Tannir M. Assessment of burnout in medical undergraduate students in Riyadh, Saudi Arabia. BMC medical education. 2019 Dec;19(1):1-8.
2. Dyrbye LN, Thomas MR, Shanafelt TD. Systematic review of depression, anxiety, and other indicators of psychological distress among U.S. and Canadian medical students. Academic Medicine. 2006.
3. Maslach C, Leiter MP. Understanding the burnout experience: Recent research and its implications for psychiatry. World Psychiatry. 2016
4. Ludwig AB, Burton W, Weingarten J, Milan F, Myers DC, Kligler B. Depression and stress amongst undergraduate medical students. BMC medical education. 2015 Dec;15(1):1-5.
5. Pacheo M, Candaras G, Welch J. Legislative Hearing Before the State Senate Committee on Global Warming and Climate Change. Western New England University, School of Law; 2017.
6. Backović D, Jevtić M. [Burnout syndrome as a mental health problem among medical students]. Med Pregl. 2012
7. Hatch PH, Winefield HR, Christie BA, Lievaart JJ. Workplace stress, mental health, and
