# Supplementary material for: Prevalence and associated risk factors of burnout amongst veterinary students in Ghana
Source: PLoS One. 2022 Jul 14;17(7):e0271434. doi: 10.1371/journal.pone.0271434 (PMC9282513; doi:10.1371/journal.pone.0271434)
Supplement: Attachment — Submitted filename: RESPONSE TO REVIEWERS(1)-1.docx [file pone.0271434.s003.docx]

31^st^ July, 2021

Manuscript PONE-D-20-27094

Staff Editor

PLOS ONE

Dear Avanti Dey (PhD),

Thank you for giving us the opportunity to submit a revised draft of the manuscript “PREVALENCE AND ASSOCIATED RISK FACTORS OF BURNOUT AMONGST VETERINARY STUDENTS IN GHANA” for publication in PLOS ONE. We appreciate the time and effort that you and the reviewers dedicated to providing feedback on our manuscript and are grateful for the insightful comments on and valuable improvements to our paper. We have incorporated most of the suggestions made by the reviewers. Those changes are highlighted within the manuscript. Please see below, in blue, for a point-by-point response to the editor’s and reviewers’ comments and concerns.

**Reviewers' Comments to the Authors:**

**Reviewer 1**

1. Table 2 indicate exercise hours if per day or week as well as sleeping hours
   Works “while” and not whiles
   Demographic characteristics and burnout 7 (70%) as well as the rest of the percentages in the same paragraph.
   Discussion and not discussion
   use abbreviations after being defined

**Authors’ response:** The reviewer is correct, and we have made the necessary grammatical error corrections as indicated. The frequency of exercise and sleeping hours have been specified in our revised manuscript.

2. In the conclusion, burnout was noted due to high depersonalization so remove low levels of exhaustion and personal achievement.

I highly recommend rewriting the conclusion as it doesn’t reflect the strengths, weaknesses and recommendations deduced from this study.

**Authors’ response:** The recommendation given has been considered and the authors have made changes to the conclusion to reflect the strengths, weaknesses and recommendations as indicated by the reviewer.

**Reviewer 2**

1. In the Method section (p. 5), I am wondering how the researchers resolved the issue of the discrepancy between what was needed for Yamane formula and what was actually obtained? This limitation might be worth discussing in more detail in the discussion section (along with any other limitations of the study).

**Authors’ response**: The cause for the discrepancy observed in the actual sample size used and that obtained from the Yamane Formula have been explained in detail in the Method section under the heading “Sample size”.

2. On pages 12 – 18, there are very limited descriptions of the statistics tests that were conducted, and the results of these tests are not well described. To remedy, I would suggest describing which tests were conducted at the outset of a paragraph or section (e.g., chi-square or logistic regression) and then include more statistical details for each of the tests (e.g., for a logistic regression you would report the overall model statistics and then individual coefficients, p values, and odds ratios).

**Authors’ response**: The detailed descriptions of the statistics tests have been previously been spelt out in the Method section under the subsection “Data Analysis” where all tests have been spelt out and what they were used for. In addition, the results on the logistic regression and chi-square tests have been incorporated in the results section as recommended by the reviewer.

3. In the discussion and/or the introduction section, can you provide specific percentages of the average rates of EE, RFA, and DP for the reader to make comparisons between the sample in this study and the larger population.

Authors’ response: The specific percentages of the average rates of EE, RFA and DP have been included in the revised manuscript.

4. The authors state on p. 18 that “burnout is expressed the most due to depersonalization.” This doesn’t seem entirely accurate given that two of the Burnout components were actually lower than.

Authors’ response: The authors have revised this expression and have rectified and rephrased the statement to reflect the true findings of this study.

5. On p. 19, the discussion about why depersonalization is higher in this sample seems underdeveloped. I would like to see the authors explore the possible reasons for this manifestation of Burnout (and not the others) in the context of this population and the stressors and life experiences they have.

**Authors’ response**: The recommendation given has been considered and the authors have developed the said deficit in writing that you have stated. More reasons have been adduced to why depersonalization was higher in this study.

6. The finding that sleep may result in tiredness and a lack of productivity doesn’t seems quite simple and not particularly novel. I’m wondering if the authors can expand on the implications for this connection as it relates to students and practicing vets

**Authors’ response:** The connection between sleep and tiredness might not be novel but in our study, however, it adds on to the substantive argument of some the source of stresses that could have easily contribute to burnout in veterinary students. This study sought to explore the risk factors associated with burnout and so on a broader view the authors thought of taking a look at how long students are able to get enough rest as a suggestive predictor of burnout.

7. The statement that “burnout was observed” at the outset of the conclusion section seems somewhat misleading because you only found that one component of Burnout was observed and in fact the other two components were actually higher than average. I would suggest that this is rephrased here to capture the nuances of the results and, again, would suggest expanding on how and why depersonalization appears to the form that burnout takes with this group.

**Authors’ response**: The recommendation given has been considered and the authors have rephrased the conclusion to reflect the findings of this study and not to mislead.
