# Supplementary material for: Prevalence and associated risk factors of burnout amongst veterinary students in Ghana
Source: PLoS One. 2022 Jul 14;17(7):e0271434. doi: 10.1371/journal.pone.0271434 (PMC9282513; doi:10.1371/journal.pone.0271434)
Supplement: Attachment — Submitted filename: RESPONSE TO REVIEWER burn out.docx [file pone.0271434.s004.docx]

**RESPONSE TO REVIEWER’S COMMENTS ON PREVALENCE AND ASSOCIATED RISK FACTORS OF BURNOUT AMONGST VETERINARY STUDENTS IN GHANA**

**Comment 1:** In the Method section (p. 5), I am wondering how the researchers resolved the issue of the discrepancy between what was needed for Yamane formula and what was actually obtained? This limitation might be worth discussing in more detail in the discussion section (along with any other limitations of the study).

**Response:** The discrepancy between the sample size used and the actual sample size obtained from the Yamane formula has been explained in the methodology aspect under the sample size section in the reviewed manuscript.

**Comment 2:** On pages 12 – 18, there are very limited descriptions of the statistics tests that were conducted, and the results of these tests are not well described. To remedy, I would suggest describing which tests were conducted at the outset of a paragraph or section (e.g., chi-square or logistic regression) and then include more statistical details for each of the tests (e.g., for a logistic regression you would report the overall model statistics and then individual coefficients, p values, and odds ratios).

**Response:** This suggestion by the reviewer has been considered by the authors. The statistical tests conducted have been described in details in the data analysis section in the reviewed manuscript.

**Comment 3:** In the discussion and/or the introduction section, can you provide specific percentages of the average rates of EE, RFA, and DP for the reader to make comparisons between the sample in this study and the larger population.

**Response:** The specific percentages of the average rates of EE, RFA, and DP have been provided appropriately to enhance comparison between the sample in this study. This has been added in the new manuscript under the discussion section.

**Comment 4:** The authors state on p. 18 that “burnout is expressed the most due to depersonalization.” This doesn’t seem entirely accurate given that two of the Burnout components were actually lower than.

**Response:** The statement “burnout is expressed the most due to depersonalization” has been looked at. The write-up in that section has been re-written to reflect the findings on the burnout components in this study. This has been highlighted in the revised manuscript with tracked changes.

**Comment 5:** On p. 19, the discussion about why depersonalization is higher in this sample seems underdeveloped. I would like to see the authors explore the possible reasons for this manifestation of Burnout (and not the others) in the context of this population and the stressors and life experiences they have.

**Response:** The authors have acknowledged the concern of the reviewer. In view of this, authors have provided possible reasons for depersonalization being the manifestation of burnout amongst the students in this study. This adjustment is found in the discussion section in the revised manuscript with tracked changes.

**Comment 6:** The finding that sleep may result in tiredness and a lack of productivity doesn’t seems quite simple and not particularly novel. I’m wondering if the authors can expand on the implications for this connection as it relates to students and practicing vets.

**Response:** The authors have provided further explanations on sleep affecting productivity and the academic performance of students and practicing veterinarians but rather focusing more on veterinary students.

**Comment 7:** The statement that “burnout was observed” at the outset of the conclusion section seems somewhat misleading because you only found that one component of Burnout was observed and in fact the other two components were actually higher than average. I would suggest that this is rephrased here to capture the nuances of the results and, again, would suggest expanding on how and why depersonalization appears to the form that burnout takes with this group.

**Response:** The conclusion has been recaptured and in the revised manuscript to capture the nuances of the results and have slightly expanded on how and why depersonalization appears to the form that burnout takes in this study.
