# Supplementary material for: Prevalence and associated risk factors of burnout amongst veterinary students in Ghana
Source: PLoS One. 2022 Jul 14;17(7):e0271434. doi: 10.1371/journal.pone.0271434 (PMC9282513; doi:10.1371/journal.pone.0271434)
Supplement: Attachment — Submitted filename: reviewer comments.docx [file pone.0271434.s001.docx]

English editing

Table 2 indicate exercise hours if per day or week as well as sleeping hours

Works “while” and not whiles

Demographic characteristics and burnout 7 (70%) as well as the rest of the percentages in the same paragraph.

Discussion and not discussion

use abbreviations after being defined

In the conclusion, burnout was noted due to high depersonalization so remove low levels of exhaustion and personal achievement.

I highly recommend rewriting the conclusion as it doesn’t reflect the strengths, weaknesses and recommendations deduced from this study.
